# Utility of Procalcitonin (PCT) and Mid regional pro-Adrenomedullin (MR-proADM) in risk stratification of critically ill febrile patients in Emergency Department (ED). A comparison with APACHE II score

**DOI:** 10.1186/1471-2334-12-184

**Published:** 2012-08-08

**Authors:** Francesco Travaglino, Benedetta De Berardinis, Laura Magrini, Cristina Bongiovanni, Marcello Candelli, Nicolò Gentiloni Silveri, Jacopo Legramante, Alberto Galante, Gerardo Salerno, Patrizia Cardelli, Salvatore Di Somma

**Affiliations:** 1 Emergency Department Azienda Ospedaliera Sant’Andrea, School of Medicine and Psychology “Sapienza” Univesity, Rome, Italy; 2 Emergency Department Policlinico A. Gemelli Catholic, University of the Sacred Heart, Rome, Italy; 3 Emergency Department Policlinico Tor Vergata, School of Medicine “Tor Vergata” Univesity, Rome, Italy; 4 Clinical and Molecular Medicine Department Azienda Ospedaliera Sant’Andrea, School of Medicine and Psychology “Sapienza” Univesity, Rome, Italy

**Keywords:** Procalcitonin, Mid regional pro-Adrenomedullin, Fever, APACHE II score

## Abstract

**Background:**

The aim of our study was to evaluate the prognostic value of MR-proADM and PCT levels in febrile patients in the ED in comparison with a disease severity index score, the APACHE II score. We also evaluated the ability of MR-proADM and PCT to predict hospitalization.

**Methods:**

This was an observational, multicentric study. We enrolled 128 patients referred to the ED with high fever and a suspicion of severe infection such as sepsis, lower respiratory tract infections, urinary tract infections, gastrointestinal infections, soft tissue infections, central nervous system infections, or osteomyelitis. The APACHE II score was calculated for each patient.

**Results:**

MR-proADM median values in controls were 0.5 nmol/l as compared with 0.85 nmol/l in patients (*P* < 0.0001), while PCT values in controls were 0.06 ng/ml versus 0.56 ng/ml in patients (P < 0.0001). In all patients there was a statistically significant stepwise increase in MR-proADM levels in accordance with PCT values (*P < 0.0001*). MR-proADM and PCT levels were significantly increased in accordance with the Apache II quartiles (*P < 0.0001 and P = 0.0012* respectively).

In the respiratory infections, urinary infections, and sepsis-septic shock groups we found a correlation between the Apache II and MR-proADM respectively and MR-proADM and PCT respectively. We evaluated the ability of MR-proADM and PCT to predict hospitalization in patients admitted to our emergency departments complaining of fever. MR-proADM alone had an AUC of 0.694, while PCT alone had an AUC of 0.763. The combined use of PCT and MR-proADM instead showed an AUC of 0.79.

**Conclusions:**

The present study highlights the way in which MR-proADM and PCT may be helpful to the febrile patient’s care in the ED. Our data support the prognostic role of MR-proADM and PCT in that setting, as demonstrated by the correlation with the APACHE II score. The combined use of the two biomarkers can predict a subsequent hospitalization of febrile patients. The rational use of these two molecules could lead to several advantages, such as faster diagnosis, more accurate risk stratification, and optimization of the treatment, with consequent benefit to the patient and considerably reduced costs.

## Background

Fever is a common symptom in the Emergency Department (ED), and it is highly suggestive of microbial infection [1]. Accurate identification of fever aetiology in patients presenting to the ED is a desirable objective, but often it is unattainable, largely because signs and symptoms of bacterial and viral infections considerably overlap each other and often are nonspecific. This leads to a delay in establishing a fast aetiological diagnosis in the ED and to an inappropriate use of antibiotics [2]. Usually, the estimation of the bacterial infections’ severity is primarily based on the presence of some characteristics suggestive of Systemic Inflammatory Response Syndrome (SIRS) as defined by the American College of Chest Physicians/Society of Critical Care Medicine Consnensus Conference, 1992[3]. This, however, may not be apparent when the patient is seen in the very first hours of the illness.

Also, this syndrome is not specific enough to distinguish infectious from non-infectious causes of inflammation, because by definition SIRS parameters can be altered in any inflammatory condition. Distinguishing between various causes of fever is based upon a combination of clinical parameters as well as laboratory values, including C-reactive protein (CRP) and leukocyte count [4]. In addition to these, Procalcitonin (PCT) has been suggested to be a novel infection biomarker [5]. Medical literature has demonstrated that in critically ill patients with sepsis, PCT is superior to CRP in diagnosing bacterial infections [6,7]. Another molecule studied in febrile and septic patients is Adrenomedullin (ADM), a 52 amino acids peptide with immune modulating, metabolic, and vasodilator activity. Its widespread production in the tissues helps to maintain a blood supply in every organ. Moreover ADM has a bactericidal activity and could be helpful in the evaluation of sepsis’ diagnosis and prognosis and in monitoring such conditions [8]. The Mid Regional fragment of pro–Adrenomedullin (MR-proADM), included between amino acids 45-92, is the more stable part of the ADM, and it has been detected in plasma of patients with septic shock as a consequence of the ADM active peptide degradation [9].

Febrile critically ill patients are a challenge for the emergency physician. It is crucial to make an accurate diagnosis as soon as possible and to risk stratify these patients in order to start prompt and appropriate treatment and to define their disposition.

Currently, no data are available on the combined use of PCT and MR-proADM in risk stratification of febrile patients admitted to the ED [10]. Few data are available on the use of PCT only in the ED [11].

The aim of our study was to evaluate the prognostic value of MR-proADM and PCT levels in a cohort of well-defined febrile patients in the ED in comparison with a disease severity index score, the Acute Physiology And Chronic Health Evaluation (APACHE II) score. This is a prognostic score used to predict mortality of critically ill patients, obtained by combining several parameters detected during the first hours after the admittance in a critical care setting. When used together with an accurate description of disease, it can prognostically stratify acutely ill patients [12].

We also evaluated the ability of MR-proADM and PCT to predict hospitalization of patients who arrive at the ED with fever.

## Methods

We enrolled 128 consecutive febrile critically ill patients admitted to the ED of three teaching hospitals in Rome, Italy (Sant’Andrea Hospital, Agostino Gemelli Hospital, and Tor Vergata Hospital). The study was approved by Sant’Andrea Hospital Ethics Committee, Agostino Gemelli Hospital Ethics Committee, and Tor Vergata Hospital Ethics Committee. All patients provided written informed consent. This was an observational, multicentric study with an enrollment period of 12 months (from October 2009 to October 2010). Patients referred to the ED with fever (body temperature > 37 °C) and a suspicion of severe infection such as sepsis, lower respiratory tract infections, urinary tract infections, gastrointestinal (GI) infections, soft tissue infections, central nervous system (CNS) infections, or osteomyelitis were sequentially recruited. Patients younger than 18 years old were excluded. After admittance to the emergency room, each patient was clinically examined by the emergency physician. Blood sampling and radiological exams were performed in accordance with guidelines [13]. For each patient a blood sample for PCT and MR-proADM was collected. The physician was blinded about the results of both the biomarkers before starting any treatment. Blood was obtained from peripheral venous catheters. The blood was separated into plasma immediately after sampling, and aliquots of these samples were stored at -80 °C and then analyzed.

MR-proADM and PCT were measured in 50 ul of plasma by a Time-Resolved Amplified Cryptate Emission (TRACE) technology assay, using kits designed for automated sandwich immunofluorescent assay of MR-proADM and PCT, respectively (KRYPTOR; BRAHMS AG). The KRYPTOR MR-proADM and PCT assays have a detection range of 0.05–100 nmol/L and 0.02-5000 ng/mL, respectively.

For each patient the emergency physician calculated the APACHE II score and completed a standardized case report form, including history, co-morbidity, vital signs, physical examination, and putative source of infections. The form ended with the emergency physician’s diagnostic suspicion, any antibiotic prescription, and the patient’s course.

A control group was considered for the comparison of PCT and MR-ProADM values between patients and the control group itself, assuming that high values of both molecules could be useful in diagnosing an infectious source in the febrile patients admitted to the ED. The control group was made up of 40 healthy volunteers, older than 18 years, who had no past medical history and were not on medication.

### Statistical analysis

Continuous variables are expressed as mean ± standard deviation (SD) or median with interquartile range (IQR) in parenthesis, unless stated otherwise. Statistical analysis was performed using Graph Pad Prism 5.0 (GraphPad Software Inc., San Diego, CA, USA), SPSS V.18 (© Copyright IBM Corporation) and SigmaPlot V.1 1 (Copyright © 2008 Systat Software, Inc.).

Two-group non-parametric comparisons were calculated by the Mann–Whitney *U* test. For multigroup comparisons, the Kruskal–Wallis test was used. Correlation analyses were performed using Spearman rank correlation. All statistical tests were two-tailed, and *P* < 0.05 was considered statistically significant.

The APACHE II score was calculated with data obtained at admittance and was expressed as percentage of predicted mortality (Predicted Death Rate = e^Logit^/(1 + e^Logit^); Logit = -3,517 + (APACHE II) * 0,146) [12].

To determine the ability of the two biomarkers to predict hospitalization, a receiver operating characteristic (ROC) curve was calculated.

## Results

Patients’ characteristics are shown in Table 1.

**Table 1:** Patient’s characteristics

**A**						
	**ALL PATIENTS**			**CONTROL**		
**GENDER**	68 MALE – 60 FEMALE			23 MALE – 17 FEMALE		
	**MEAN ± SD**	**MEDIAN (IQR)**	**RANGE (MIN-MAX)**	**MEAN ± SD**	**MEDIAN (IQR)**	**RANGE (MIN-MAX)**
**AGE (Y)**	61 ± 19	65 (45-77)	18-96	55 ± 15	58.5 (45-70)	29-76
**TEMPERATURE (°C)**	38.7 ± 0.7	38.6 (38.1-39)	37-41	36.4 ± 0.3	36.4 (36.1-36.5)	36-36,7
**B**						
**PCT (ng/ml)**	3.85 ± 7.63	0.56* (0.10-3.44)	0.02-41.06	0.06 ± 0.02	0.06* (0.04-0.08)	0.02-0.11
**MR-ProADM (nmol/ml)**	1.72 ± 0.10	0.85** (0.50-1.68)	0.05-15.3	0.50 ± 0.10	0.50** (0.40-0.58)	0.31-0.65
**APACHE II (%)**	13.5 ± 11.21	9.9 (6.70-15.50)	2.9-76			

*p < 0.0001.

**p < 0.0001.

### MR-proADM and PCT

**Figure 1:**
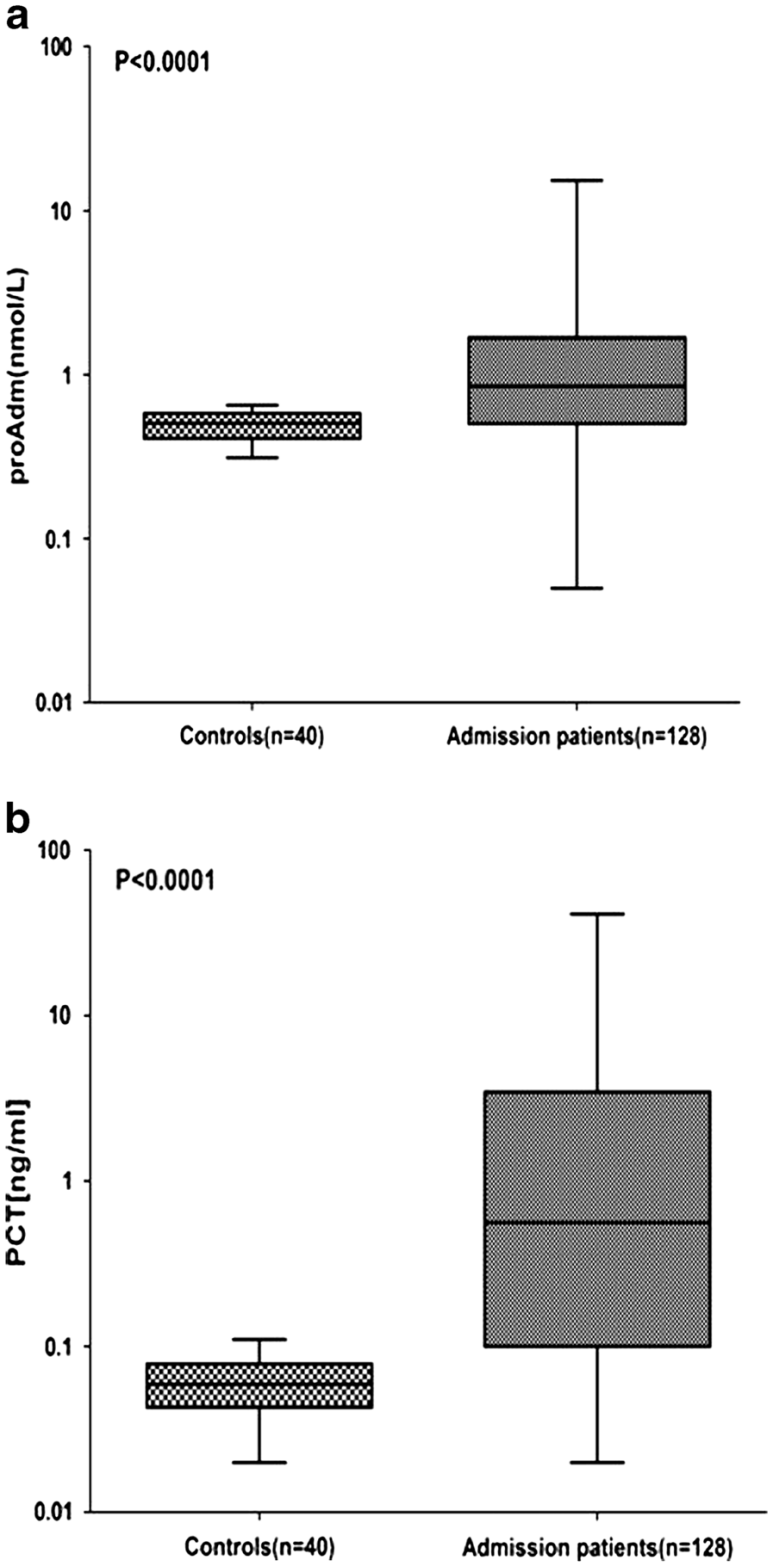
**Levels of MR-proADM and PCT at admission.** (**a**) MR-proADM in all patients versus healthy control individuals. (**b**) PCT in all patients versus healthy control individuals. Lines denote median values, boxes represent 25–75th percentiles and whiskers indicate the range. The numbers of samples are indicated in parentheses. proADM: mid-regional pro-adrenomedullin; PCT: procalcitonin.

MR-proADM and PCT values were compared between healthy subjects (control group) and patients (Figure 1a, b). MR-proADM median (range) values in controls were 0.5 nmol/l (0.40–0.58 nmol/l) as compared with 0.85 nmol/l (0.50–1.68 nmol/l) in patients (*P* < 0.0001), while PCT values in controls were 0.06 ng/ml (0.04–0.08 ng/ml) versus 0.56 ng/ml (0.1–3.4 ng/ml) in patients (P < 0.0001) (Table 1b).

**Figure 2:**
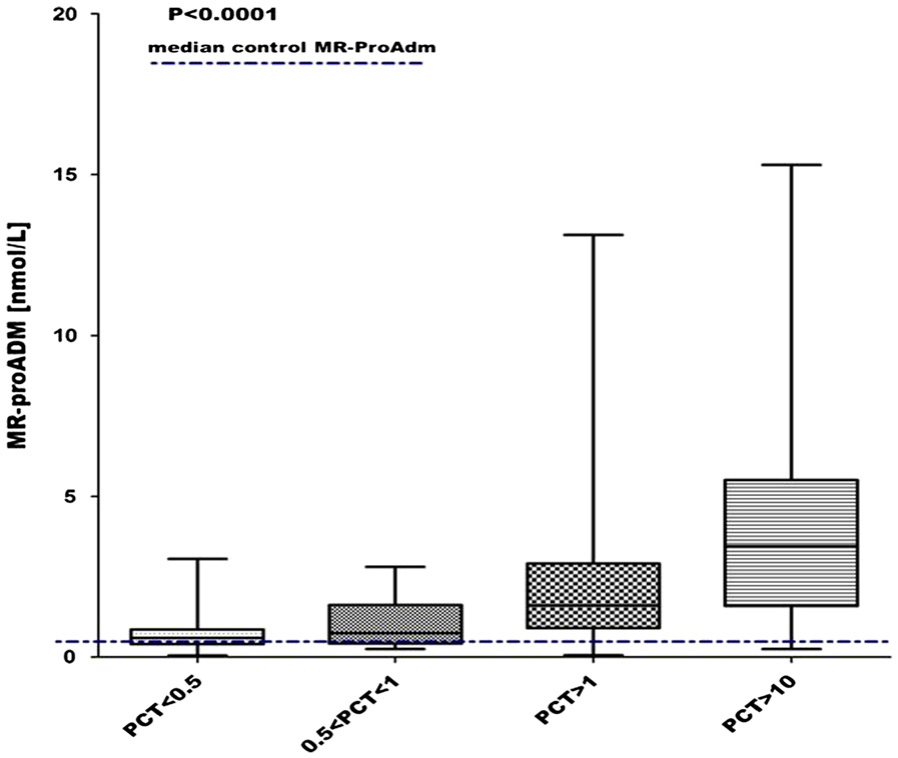
**Correlation between MR-proADM and PCT.** MR-proADM levels increase in according to PCT values in all patients. The dashed line represents the median in controls.

MR-proADM levels showed a correlation with PCT levels in the patients’ group (r = 0.57, P < 0.0001). In all patients there was a statistically significant stepwise increase in MR-proADM levels in accordance with PCT values (*P < 0.0001* - Figure 2).

According to the final diagnosis made in the ED, the patients were divided into five different groups. The principal diagnosis and relative groups are summarized in Table 2.

**Table 2:** Clinical diagnosis of patients

**Group**	**Total patients**	**Diagnosis**	**Number of patients**	**MR-ProADM nmol/ml median(IQR)**	**PCT ng/ml median(IQR)**	**Apache II median(IQR)**
**Fever**	19	Unknown origin	19	0.54 (0.40-0.70)	0.25 (0.08-0.63)	6.70 (5.10-8.70)
**Respiratory**	44	Pneumonia	31	0.90 (0.49-1.65)	0.48 (0.07-1.64)	
		Bronchitis	5			
		Pleural effusion	1			
		COPD	6			
		TB	1			
**Urinary**	27	Urinary infection	27	0.86 (0.51-1.68)	1.32 (0.07-3.89)	10.60 (6.30-16.50)
**Sepsis**	20	Sepsis	18	1.82 (0.70-5.72)	2.49 (0.24-16.51)	
		Septic shock	2			
**Other**	18	Endocarditis	4	0.89 (0.53-1.9)	0.39 (0.16-4.21)	
		Necrotizing	3			
		Fasciitis				
		Gangrene	2			
		Pericarditis	6			
		Gastroenteritis	3			

COPD: chronic obstructive pulmonary disease; TB: tuberculosis.

### APACHE II score analysis

**Figure 3:**
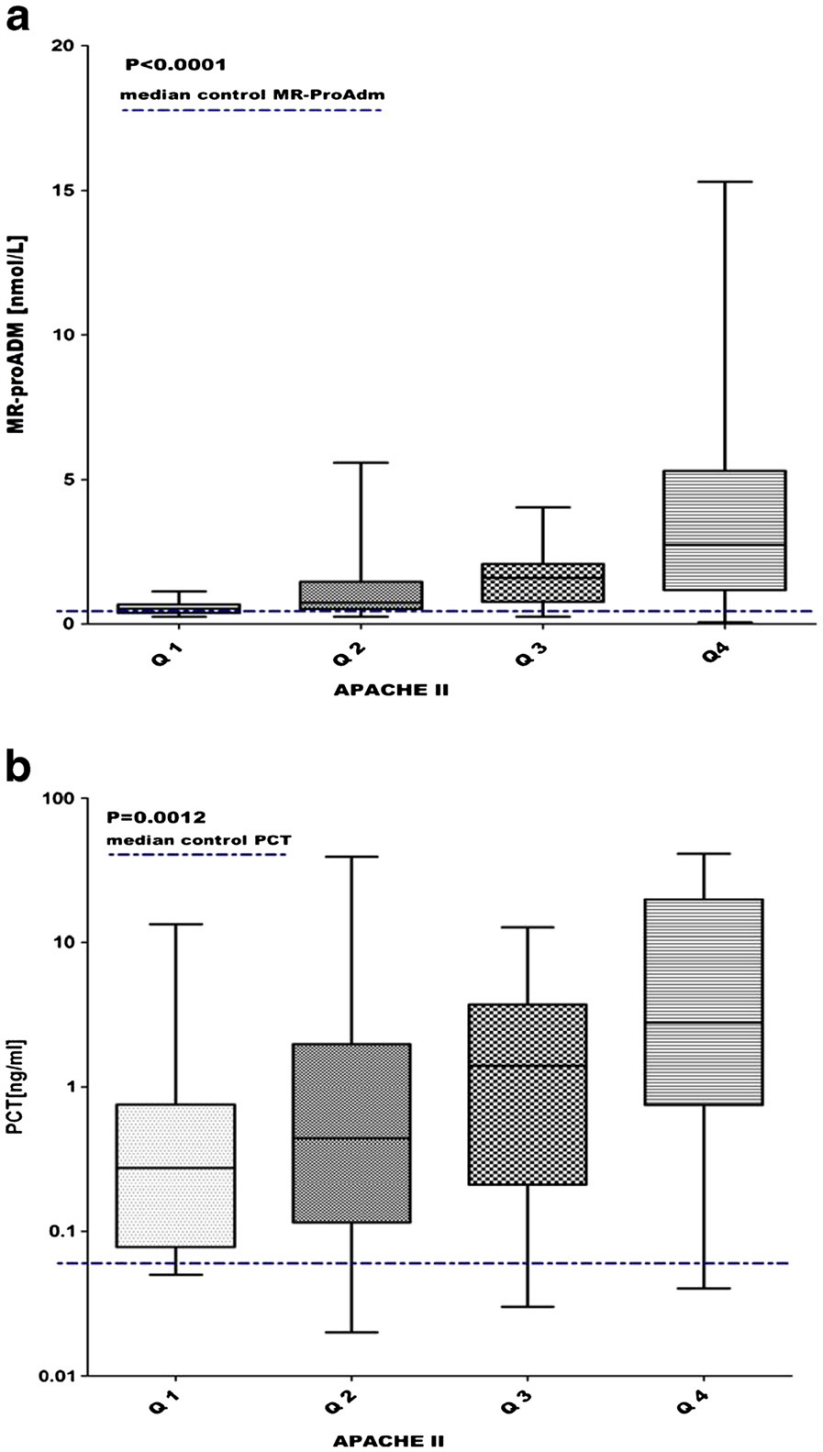
**Correlation between APACHE II quartiles and MR-proADM (fig. 3a) and PCT (fig. 3b).** 3**a**) Apache II quartiles (Q1-4) vs MR-proADM levels: Q1 0.54 (0.39-0.68), Q2 0.75 (0.53-1.50), Q3 1.61 (0.67-2.08), Q4 2.75 (1.18-5.30). 3b) Apache II quartiles (Q1-Q4) vs PCT levels: Q1 0.27 (0.08-0.75), Q2 0.44 (0.11-1.98), Q3 1.40 (0.21- 3.69), Q4 2.79 (0.75-19.74). Lines denote median values, boxes represent 25–75th percentiles and whiskers indicate the range.

The APACHE II score, expressed in predicted mortality percentage, was grouped into quartiles and compared respectively with MR-proADM and PCT levels. MR-proADM and PCT levels were significantly stepwise increased in accordance with the APACHE II quartiles (*P < 0.0001 and P = 0.0012* respectively) (Figure 3a and b).

Furthermore, the correlation between MR-proADM and PCT in each APACHE II quartile group was tested. A positive correlation was shown in quartile 2 (r = 0.64, P = 0.0005), quartile 3, (r = 0.53, P = 0.006) and quartile 4 (r = 0.53, P = 0.008). No correlation was found in quartile 1.

In all the patients, the correlation between the APACHE II score and MR-proADM was significant with a p value < 0.0001 and r = 0.39, while the correlation between the APACHE II score and PCT was significant with a p value = 0.003 and r = 0.30.

Moreover, the correlation between the APACHE II score and the two biomarkers MR-proADM and PCT was tested in the following groups of patients, divided according to the final ED’s diagnosis: respiratory infections group, urinary infections group, sepsis and septic shock group, unspecified fever group, and “other” group (Table 2).

In the respiratory infections group a positive correlation was found between the APACHE II and MR-proADM (r = 0.66, P = 0.0002) and between MR-proADM and PCT(r = 0.54, P = 0.0001). The urinary infections group showed a positive correlation between the APACHE II and MR-proADM (r = 0.50, P = 0.001) and between MR-proADM and PCT(r = 0.61, P = 0.0007). In the sepsis-septic shock group a positive correlation was found between the APACHE II and MR-proADM (r = 0.66, P = 0.02) and the APACHE II and PCT (r = 0.65, P = 0.02). In the remaining groups, no statistically significant correlations were identified.

### Hospitalization prediction

**Figure 4:**
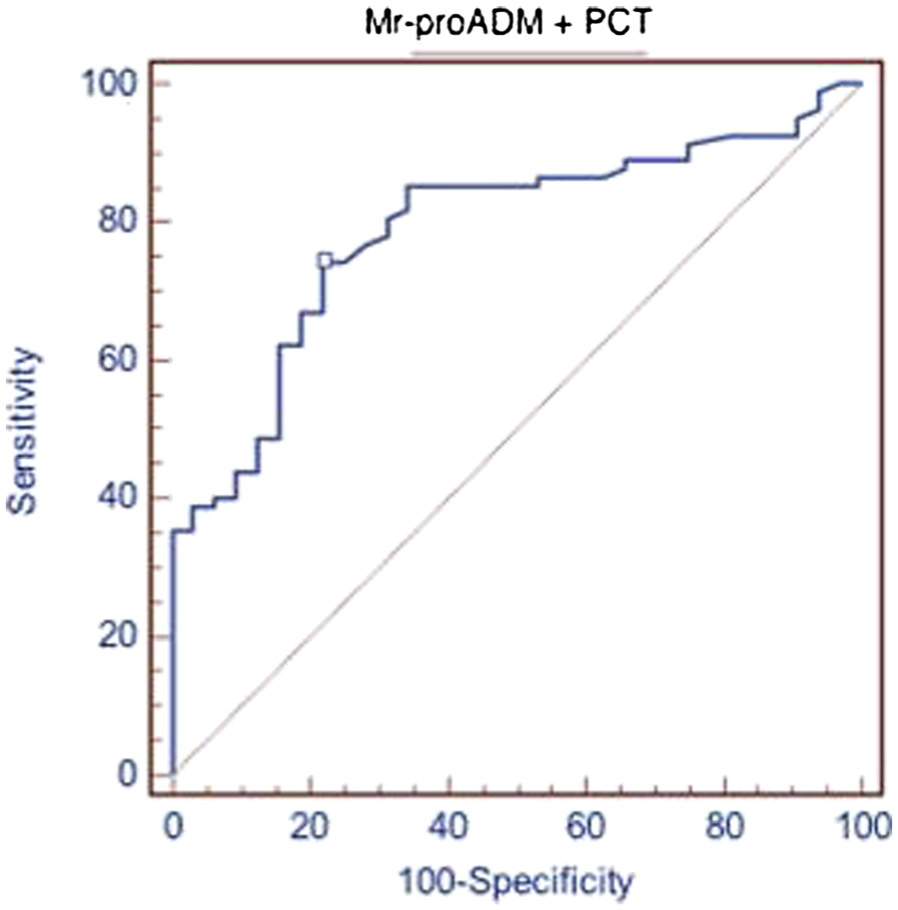
**Combined used of MR-proADM plus PCT in predicting hospitalization.** Area under the ROC curve (AUC) 0,79; standard error 0,0426, 95 % Confidence interval 0,704 to 0,861; significance level P (Area = 0.5) 0,0001.

We evaluated the ability of MR-proADM and PCT to predict hospitalization in patients admitted to our emergency departments complaining of fever. The patients hospitalized after ED admittance were 99. MR-proADM alone had an AUC of 0.694 (p = 0,0002), while PCT alone had an AUC of 0.763 (p = 0,0001). The combined use of PCT and MR-proADM instead showed an AUC of 0.79 (p = 0,0001). Results are summarized in Figure 4.

## Discussion

In the present study we attempted to evaluate whether MR-proADM and PCT could play a relevant role in decision making for the disposition of the febrile patients in the ED. We tried to understand the real diagnostic and prognostic weight of the two biomarkers for the emergency physician in taking care of a critical febrile patient.

First we tested the diagnostic relevance of infection for the two biomarkers, comparing the values detected in patients with those of healthy control individuals. According to literature [6-8], our data confirmed higher values of both biomarkers in febrile patients compared to healthy controls (Table 1b).

PCT added diagnostic value to the currently routine variables (CRP, WBC count) for the identification of infection in patients with fever. A negative value of PCT should be of relevance in ruling out the presence of infections, which is helpful when choosing the admitting ward and/or antibiotic treatment [14].

Concerning the prognostic role of MR-proADM and PCT, we evaluated the ability of MR-proADM and PCT in predicting the subsequent hospitalization of patients who arrived at the ED complaining of fever. Our data demonstrated a better predictive value, even slightly, for PCT than MR-proADM (AUC of 0.763 and a *p* =0, 0001 versus an AUC of 0.694 and a *p* =0, 0002, respectively). The hospitalization’s predictive value rose if the two biomarkers were used together (AUC of 0.79 and *p* =0, 0001 – Figure 4).

Focusing on data emerging from this study, we can highlight that MR-pro ADM and PCT levels are elevated in critically ill febrile patients, and that they seem to have a prognostic value similar to the APACHE II score, providing an additional margin of safety. Thus, we evaluated the potential correlation between the two molecules and the APACHE II score, to determine the possible additional value of PCT and MR-proADM in predicting the outcome of the febrile patient attending the ED [14-18]. The APACHE II score has been expressed in predicted mortality percentage.

The Apache II score was grouped into quartiles and compared with levels of MR-proADM and PCT. MR-proADM and PCT levels were significantly stepwise increased in accordance with the Apache II quartiles with a *p* value *< 0.0001 and = 0.0012* respectively (Figure 3a, b), proving that a higher value of MR-proADM and of PCT corresponds to a higher APACHE II score.

A positive correlation between the APACHE II score and both biomarkers was found in all patients, considered in their globality, with a *p <0.0001* for MR-proADM and a *p =0.003* for PCT.

Although a direct correlation between MR-proADM and PCT and mortality cannot be shown, the correlation between the two biomarkers and the APACHE II score seems to assert their prognostic role in the febrile patient attending the ED, suggesting that MR-proADM may predict mortality in a larger number of clinical conditions, compared to PCT. Moreover, we found a stepwise increase of MR-proADM levels with PCT values (*P < 0.0001* - Figure 2).

To date, PCT is described as a marker of bacterial infection, but it has also been proposed and identified as a mortality predictor in patients with community-acquired pneumonia, in critically ill patients with sepsis, and in those with ventilator associated pneumonia [19-23]. In order to support this, several studies have demonstrated the diagnostic role of PCT in detecting bacterial infection and sepsis [14,24-26], and further papers have investigated its prognostic value [27-33]. In this perspective, our data suggest that the positive correlation between PCT and the APACHE II score in febrile patients who arrive in the ED correspond to the prognostic value of the APACHE II score [34-37]. Moreover our data show that higher levels of PCT are related to more-severe infections and worse prognosis, as evidenced by the following division of the patients into 5 groups based on the clinical diagnosis made in the ED: respiratory infections group, urinary infections group, sepsis and septic shock group, unspecified fever group, and “other” group (Table 2). In patients so grouped, a positive correlation between the APACHE II score and MR-proADM was found in the respiratory infections group (r = 0.66, P = 0.0002), in the urinary infections group (r = 0.50, P = 0.001) and in the sepsis and septic shock group (r = 0.66, P = 0.02). For PCT, the positive correlation with the APACHE II score was found only in the sepsis and septic shock group (r = 0.65, P = 0.02).

We can also suggest that the use of these biomarkers in the febrile patient could not only improve diagnostic and prognostic accuracy, but could also promote a rational use of antibiotics, in terms of reduction of antibiotic overconsumption and adverse events as shown in recent studies that point to the effectiveness of PCT in safely reducing the number of unnecessary antibiotic prescriptions [2,38-45].

## Conclusions

The present study has highlighted how MR-proADM and PCT may be helpful to the febrile patient’s care in the emergency department. Our data support the prognostic role of MR-proADM and PCT in that setting, as demonstrated by the correlation with the APACHE II score, particularly in respiratory tract infections, urinary tract infection, and sepsis. Also of interest is the possibility that the combined use of the two biomarkers can predict a subsequent hospitalization of febrile patients accessing the ED.

The rational use of these two molecules could lead to several advantages, such as faster diagnosis, more accurate risk stratification, and optimization of treatment, with consequent benefit to the patient.

Our study has some obvious limits, including a relatively small patient sample and lack of follow-up. It would be useful to conduct additional studies to further validate the role of both MR-proADM and PCT in febrile patients’ care in the emergency department.

### Key messages

1. Fever is one of the most frequent signs seen in emergency departments (ED) and in the critical care setting in general. It is a priority because delayed diagnosis of infection can lead to sepsis with high morbidity and mortality.

2. Modern medicine requires optimizing resources and time when evaluating patients with fever. To this end, medical literature focuses on identifying biomarkers as diagnostic and possibly prognostic indicators. Among the biomarkers that can play a role are PCT and MR-ProADM.

3. The positive correlation between these two molecules and the APACHE II score demonstrate their prognostic value, especially in selected groups of patients, such as those with respiratory and urinary tract infections and those with sepsis and septic shock.

4. The combined use of PCT and MR-ProADM can help predict the hospitalization of patients that come to the ED complaining of fever.

5. The combined use of these two biomarkers can improve the management of febrile patients in the ED and in the critical care setting in general.

## Abbreviations

ADM, Adrenomedullin; APACHE II score, Acute Physiology And Chronic Health Evaluation II score; AUC, area under the curve; CRP, C-reactive Protein; CNS, central nervous system; ED, Emergency Department; GI, gastrointestinal; IQR, interquartile range; MR-proADM, Mid regional pro-Adrenomedullin; PCT, Procalcitonin; ROC, receiver operating characteristic; SD, standard deviation; SIRS, Systemic Inflammatory Response Syndrome; SPSS, Statistical Package for the Social Sciences; TRACE, Time-Resolved Amplified Cryptate Emission; WBC, white blood cells.

## Competing interests

The authors declare that they have no competing interests.

## Authors’ contributions

FT designed the study and participated in writing the manuscript. BDB participated in the design of the study, enrolled the patients, and participated in writing the manuscript. LM participated in the design of the study. CB participated in the enrollment of the patients and in writing the manuscript. MC participated in the enrollment of the patients. NGS participated in the design of the study. JL participated in the enrollment of the patients. AG participated in the design of the study. GS carried out the immunoassays and performed the statistical analysis. PC participated in carrying out the immunoassays. SDS participated in the design of the study and revised it. All authors read and approved the final manuscript.

## Pre-publication history

The pre-publication history for this paper can be accessed here:

http://www.biomedcentral.com/1471-2334/12/184/prepub
